# Navigating Asymptomatic Idiopathic Pneumoperitoneum: A Case Report and a Literature Review

**DOI:** 10.7759/cureus.55687

**Published:** 2024-03-06

**Authors:** Rabbani Mahmoud Daoud, Salma M Daoud, Manar Almansoor, Noora F Ali, Esra S Salman

**Affiliations:** 1 School of Medicine, Royal College of Surgeons of Ireland (RCSI), Busaiteen, BHR; 2 General Practice, Albaraka Fertility Hospital, Manama, BHR; 3 School of Medicine, Royal College of Surgeons in Ireland, Busaiteen, BHR; 4 General Surgery, Salmaniya Medical Complex, Manama, BHR

**Keywords:** surgery, intra-epithelial eosinophilia, negative laparotomy, free air under the diaphragm, free air in abdomen, idiopathic spontaneous pneumoperitoneum

## Abstract

Pneumoperitoneum refers to the presence of free air in the abdominal cavity, typically indicating viscus perforation requiring urgent surgical intervention. Occasionally, pneumoperitoneum occurs without organ perforation, termed 'spontaneous’ or ‘non-surgical’ pneumoperitoneum. We present the case of a 65-year-old male referred to the emergency department after a seizure episode. The patient reported no abdominal pain or fever, and examination revealed no other signs of peritonitis. An erect chest X-ray showed air under the diaphragm, and a subsequent computed tomography (CT) scan confirmed free intraperitoneal air in the abdomen. The patient underwent a prompt exploratory laparotomy to assess for abdominal perforation, but the findings were negative. He had an uneventful post-operative hospital course and was discharged nine days after admission. This case underlines the importance of considering spontaneous idiopathic pneumoperitoneum (SIP) in asymptomatic patients and discusses management options.

## Introduction

'Pneumoperitoneum' refers to the presence of free gas in the peritoneal cavity, outside the visceral organs [1]. It is most commonly caused by a perforated hollow viscus or post-trauma [2], leading to clinical manifestations such as abdominal pain, tenderness, and signs of peritonitis [3], often requiring urgent surgical intervention. Additionally, it can be associated with surgical abdominal access in laparoscopic procedures and laparotomies. A small subset of patients, approximating 10%, may experience "spontaneous" or "on-surgical" pneumoperitoneum [4], attributed to other abdominal, thoracic, or gynecological etiologies, such as pneumatosis cystoides intestinalis (PCI) and roncho-peritoneal fistula [5,6].

Spontaneous idiopathic pneumoperitoneum (SIP) is an even rarer subtype of spontaneous pneumoperitoneum, where the presence of free air in the abdominal cavity cannot be attributed to any known cause, making it a diagnosis of exclusion [7]. In a retrospective study conducted by Peker et al. in 2017, the rate of spontaneous idiopathic pneumoperitoneum was found to be 2.36% among 338 patients with radiologically confirmed intra-abdominal free air [8].

The precise etiology of pneumoperitoneum may sporadically be difficult to identify, rendering its management and treatment challenging. Diagnostic laparoscopy is often performed to rule out perforation [9], but a definitive diagnosis may require exploratory laparotomy. Conservative management may be considered for clinically stable patients without acute abdomen findings and other signs of peritonitis [10,11].

In this report, we present a case of an incidentally detected asymptomatic spontaneous idiopathic pneumoperitoneum. The patient exhibited no signs or symptoms indicative of peritonitis, had normal inflammatory markers, and had no identifiable cause for the pneumoperitoneum on further review. We acknowledge the rarity of this case, emphasizing the necessity for careful evaluation and an individualized approach to treatment. At present, there are no established guidelines for the management of asymptomatic idiopathic spontaneous pneumoperitoneum, thereby underscoring the imperative to report instances of this rare phenomenon. This will facilitate the tailoring of management strategies for future cases and mitigate the need for unnecessary diagnostic workups and surgical interventions.

## Case presentation

A 65-year-old male was brought to the emergency room (ER) following a witnessed three-minute seizure. Upon ER arrival, the patient had fully recovered, displaying full consciousness and orientation to place, time, and person. His vital signs were recorded as follows: BP: 150/80 mmHG; RR: 18 breaths per minute; HR: 130 breaths per minute; and SaO_2_: 97% on room air. On history-taking, the patient’s chief complaint was notable for prodromal seizure symptoms, including irritability and headache, leading to a three-minute tonic-clonic seizure, followed by an unremarkable postictal state. Additionally, he reported a several-day history of constipation, dysuria, and reduced urine output. He denied any history of abdominal pain, nausea, vomiting, or fever. His past medical history was significant for hypertension, type II diabetes mellitus, epilepsy, and a previous stroke. His surgical history included a craniotomy six months ago and a kidney donation 22 years ago. The patient had no history of recent travel or sick contacts. On examination, his abdomen was soft, lax, non-tender, and not distended.

**Figure 1:**
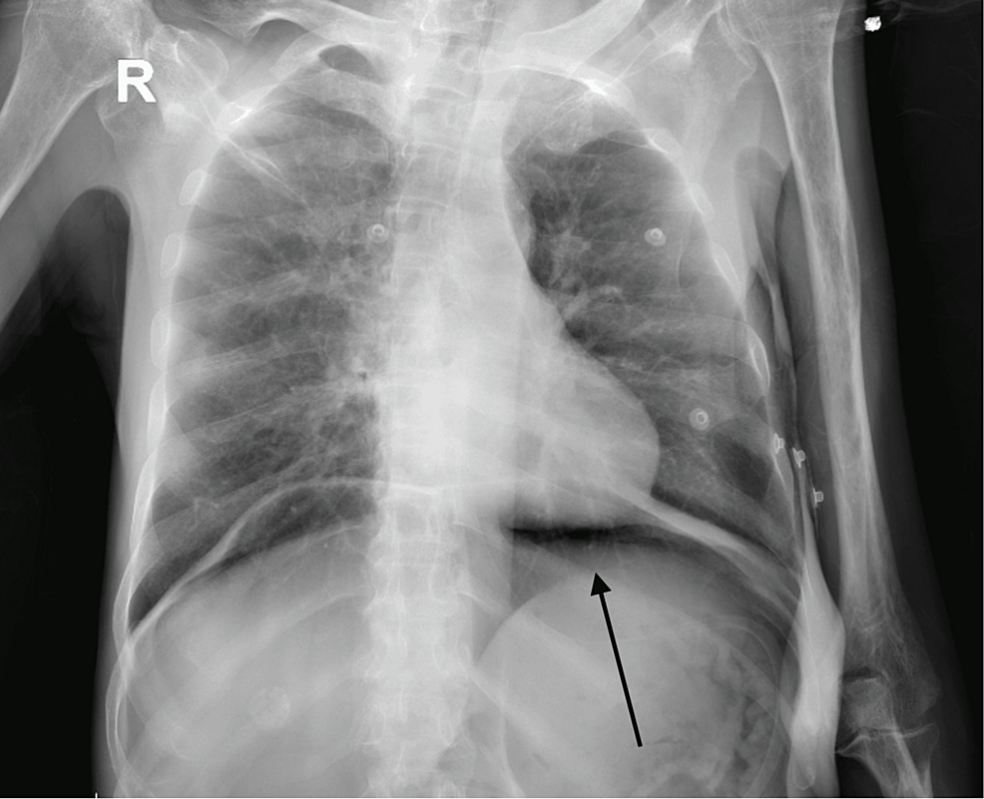
Chest X-ray of the patient on emergency room arrival.

**Figure 2:**
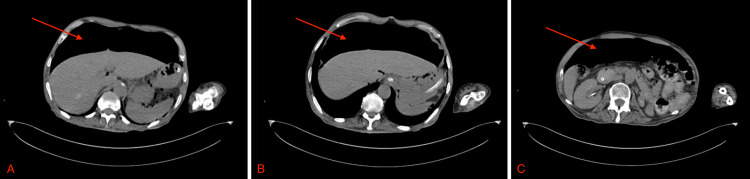
CT-scan showing massive air under the diaphragm at multiple levels, preceding the surgical intervention. CT: computed tomography.

An abdominal X-ray was initially requested in the ER to investigate the patient’s constipation, which incidentally showed a large amount of intraperitoneal free air. Subsequently, a chest X-ray also revealed a significant amount of air under the diaphragm, raising concerns for a perforated viscus (Figure 1). An immediate surgical consultation was sought, and an abdominal and pelvic computed tomography (CT) scan was ordered. The CT scan revealed extensive pneumoperitoneum in the right and left upper abdominal quadrants, without accompanying abnormal wall thickening or dilatation in the small or large bowel loops (Figure 2). It also showed a severely edematous urinary bladder with significant surrounding inflammatory changes, indicating severe cystitis. The patient’s serum laboratory results were as follows: white blood cell count (WBC) of 9.05 × 10^9^/L, hemoglobin (Hb) of 10.0 g/dL, serum C-reactive protein (CRP) of 1.2 mg/L, and otherwise unremarkable.

The patient was promptly taken to the operating theater, where he underwent an exploratory laparotomy to assess for abdominal perforation, with consideration for potential bowel resection and ostomy. The operative findings were as follows: upon entering the peritoneum, a gush of air was observed, but there were no fluids or signs of peritoneal pathology. No abnormalities were found in the small or large intestine. The stomach and duodenum were examined for perforation, and none were detected either. Furthermore, no pathology was noted in the liver, spleen, gallbladder, or appendix. Multiple enlarged lymph nodes were noted and biopsied, which revealed features of mature adipose tissues with fat necrosis and no evidence of malignancy on histopathology. A sample of peritoneal fluid was obtained and sent to the microbiology department for cytology, which was negative for microorganisms, and culture, which was sterile.

The patient was transferred to the high dependency unit (HDU), where he made a complete and uneventful recovery. Subsequently, the patient was transferred to the ward for further evaluation by the internal medicine team in order to rule out alternative causes of pneumoperitoneum before arriving at a diagnosis of SIP. This assessment involved ruling out an emphysematous lung injury or a fistula connecting the pleural and intraperitoneal cavities, suggestive of a pneumoperitoneal fistula (PPF) on chest X-rays and chest CT. Additionally, there were no clinical signs or symptoms indicative of pneumothorax, pneumomediastinum, or tuberculosis (TB), and the patient had no recent travel history. The absence of peritoneal dialysis in the patient’s medical records, as well as the lack of recent surgical procedures, ruled out post-operative and peritoneal dialysis-induced pneumoperitoneum. Lastly, laboratory tests showed no evidence of infection or inflammation that could suggest necrotizing enterocolitis or an inflammatory bowel disease. The patient was advised to take bed rest and was treated for cystitis. He was discharged upon resolution of his cystitis symptoms after spending a total of nine days as an inpatient and was given an appointment for a follow-up in one month, but has unfortunately missed his appointment.

## Discussion

Idiopathic spontaneous pneumoperitoneum is a rare condition for which there are currently no established guidelines for management and treatment. Attaining a definitive conclusion regarding the presence of perforation can be particularly challenging outside of the operating theater given the variability of the clinical presentation, ranging from asymptomatic to severe acute abdomen and other signs of peritonitis [3]. This diverse symptomatology renders the preposition for a standard treatment protocol for pneumoperitoneum based on clinical presentation hard to conceive. Sakaguchi et al. proposed an algorithm in 2021 [12] for the initial management of pneumoperitoneum, considering specific symptoms such as peritonitis, ascites, and sepsis. The algorithm entails an escalation of management options ranging from ‘careful follow-up’ to the ‘operating room.’ In another article in 2023, Sodade et al. advocated for a multispecialty approach, demonstrating its efficacy in reducing unnecessary surgical intervention, improving clinical outcomes, and leading to a more favorable prognosis [13].

Nevertheless, many cases of spontaneous pneumoperitoneum have been reported where surgical exploration was deemed necessary, yet no evidence of perforation was subsequently found. In a study by Chandler et al., the laparotomy rate among patients with spontaneous pneumoperitoneum was reported to be 28% [14]. Similarly, Mularski et al. reviewed 196 cases of spontaneous pneumoperitoneum, with 23% undergoing surgical exploration without identifying any perforated viscus [15]. More recently, Morrison et al. conducted a case series of spontaneous pneumoperitoneum from April 2011 to September 2016 at UPMC Horizon, reporting an exploratory laparotomy rate of 20% [16].

We conducted a literature review on reported cases of asymptomatic idiopathic spontaneous pneumoperitoneum across PubMed (Table 1). To the best of our knowledge, only four cases have been reported in English-language literature, comprising three males and one female, with a mean age of 70. Pneumoperitoneum was incidentally discovered during a routine medical check-up in one case, during an examination for respiratory symptoms in two cases, and during an admission for left abdominal flank pain in one case. Conservative treatment was the management of choice for two patients, while the other two were only closely monitored without intervention. The average hospital stay for all cases was five days, and follow-up evaluations were conducted at two weeks, one month, two months, and four months. In two patients, pneumoperitoneum persisted on CT scans during follow-up, although they remained asymptomatic. Three patients retained their diagnosis of idiopathic pneumoperitoneum, while one was later diagnosed with PCI during follow-up.

**Table 1:** Reported cases of asymptomatic idiopathic spontaneous pneumoperitoneum in English literature. CT: computed tomography.

Year	Author	Age	Modality	Treatment	Prognosis
2019	Hannan et al. [17]	71	CT thorax	Close observation	Recovery and discharge with follow-up
2020	Sidiqi et al. [18]	76	CTPA	Conservative treatment	
2021	Sakaguchi et al. [12]	88	CT thorax	Conservative treatment	
2022	Raabe et al. [19]	46	CT abdomen	Conservative treatment	

Several factors influenced the decision to perform exploratory laparotomy in our case. The presence of a language barrier also impeded our ability to promptly obtain a comprehensive patient history, and uncertainties persisted regarding potential trauma related to the seizure episode on presentation. Additionally, the extent of the radiologically identified pneumoperitoneum was significantly greater than that of the previously mentioned cases, which warranted a more interventional approach. It would be interesting to follow up with our patient in a few months to assess the status of his pneumoperitoneum - whether it has resolved, diminished, or even increased.

## Conclusions

In conclusion, it is crucial to distinguish pneumoperitoneum caused by viscus perforation from spontaneous pneumoperitoneum, as the latter typically does not necessitate surgical intervention. The management of pneumoperitoneum entails vigilant monitoring, repeated physical assessments, and investigation into the underlying cause. Surgical intervention is typically reserved for cases with significant abdominal symptoms or signs of peritonitis, but the surgeon may also consider surgical intervention in cases of massive pneumoperitoneum or an inconclusive clinical picture. Ultimately, the decision to pursue conservative management or surgical intervention should be based on the individual patient's clinical presentation and a multi-disciplinary approach.
